# Mortality prediction and influencing factors for intensive care unit patients with acute tubular necrosis: random survival forest and cox regression analysis

**DOI:** 10.3389/fphar.2024.1361923

**Published:** 2024-05-23

**Authors:** Jinping Zeng, Min Zhang, Jiaolan Du, Junde Han, Qin Song, Ting Duan, Jun Yang, Yinyin Wu

**Affiliations:** ^1^ Department of Epidemiology and Health Statistics, School of Public Health, Hangzhou Normal University, Hangzhou, China; ^2^ Department of Occupational and Environmental Health, School of Public Health, Hangzhou Normal University, Hangzhou, China; ^3^ Research on Accurate Diagnosis and Treatment of Tumor, School of Pharmacy, Hangzhou Normal University, Hangzhou, China; ^4^ Department of Nutrition and Toxicology, School of Public Health, Hangzhou Normal University, Hangzhou, China

**Keywords:** acute tubular necrosis, random survival forest, cox regression, nomogram, risk factors

## Abstract

**Background:** Patients with acute tubular necrosis (ATN) not only have severe renal failure, but also have many comorbidities, which can be life-threatening and require timely treatment. Identifying the influencing factors of ATN and taking appropriate interventions can effectively shorten the duration of the disease to reduce mortality and improve patient prognosis.

**Methods:** Mortality prediction models were constructed by using the random survival forest (RSF) algorithm and the Cox regression. Next, the performance of both models was assessed by the out-of-bag (OOB) error rate, the integrated brier score, the prediction error curve, and area under the curve (AUC) at 30, 60 and 90 days. Finally, the optimal prediction model was selected and the decision curve analysis and nomogram were established.

**Results**: RSF model was constructed under the optimal combination of parameters (mtry = 10, nodesize = 88). Vasopressors, international normalized ratio (INR)_min, chloride_max, base excess_min, bicarbonate_max, anion gap_min, and metastatic solid tumor were identified as risk factors that had strong influence on mortality in ATN patients. Uni-variate and multivariate regression analyses were used to establish the Cox regression model. Nor-epinephrine, vasopressors, INR_min, severe liver disease, and metastatic solid tumor were identified as important risk factors. The discrimination and calibration ability of both predictive models were demonstrated by the OOB error rate and the integrated brier score. However, the prediction error curve of Cox regression model was consistently lower than that of RSF model, indicating that Cox regression model was more stable and reliable. Then, Cox regression model was also more accurate in predicting mortality of ATN patients based on the AUC at different time points (30, 60 and 90 days). The analysis of decision curve analysis shows that the net benefit range of Cox regression model at different time points is large, indicating that the model has good clinical effectiveness. Finally, a nomogram predicting the risk of death was created based on Cox model.

**Conclusion:** The Cox regression model is superior to the RSF algorithm model in predicting mortality of patients with ATN. Moreover, the model has certain clinical utility, which can provide clinicians with some reference basis in the treatment of ATN and contribute to improve patient prognosis.

## 1 Background

Acute tubular necrosis (ATN) is the most common type of acute renal failure (accounting for approximately 70%–80%) (Zhou et al., 2010). It is a clinical syndrome caused by ischemia of renal tissue or necrosis of tubular epithelial cells due to toxic damage, and resulting in a dramatic decrease in the glomerular filtration rate (An et al., 2022). It is often manifested as progressive azotemia, electrolyte disturbance, acid-base balance disorder, and a host of other symptoms. ATN patients not only have severe renal failure, but also have many comorbidities, such as myocardial infarction, congestive heart failure, peptic ulcer disease, etc., which can be life-threatening and require timely treatment. ATN is associated with high mortality, especially for those patients in the intensive care unit (ICU) (Rosen and Stillman, 2008). Understanding the influencing factors of ATN and taking appropriate interventions can effectively shorten the duration of the disease to reduce mortality and improve patient prognosis. Previous studies have shown that pH, base excess, creatinine, and blood urea nitrogen (BUN) are common influencing factors of ATN (Liaño et al., 1989). However, other potential risk factors that may affect the prognosis of patients with ATN have not yet been identified. In recent years, the continuous development of medical information technology and the popularization of electronic medical record systems have generated a large quantity of data for prognostic model evaluation and other clinical applications.

Cox regression model is the most common semi-parametric regression model, which can analyze the influence of multiple factors on outcome events and carry out statistical analysis on data with censoring (Moolgavkar et al., 2018). This method improves the efficiency and reliability of survival analysis by considering multiple predictor variables simultaneously (Koletsi and Pandis, 2017). It has been widely used in medical research, such as evaluating the survival rate of cancer patients, the risk of heart failure, and the prognosis prediction of patients (Hippisley-Cox and Coupland, 2015; Tang M. et al., 2021; Wang et al., 2022). On the other hand, machine learning (ML) is a data-based algorithmic technology that automatically analyzes and learns patterns and regularities in data to predict and optimize future outcomes, and is a fast-growing area. ML technology has also been applied in many aspects of medical research, which is important for improving medical care and promoting human health (Noorbakhsh-Sabet et al., 2019; Issa et al., 2021). Random survival forest (RSF) is a comprehensive method of random forest and survival analysis, which processes right censored data. Different from the general binary classification method, the target variable of survival analysis method is survival time. By training a large number of survival trees, the model votes out the final prediction results weighted from individual trees in the form of voting. RSF is a ML algorithm based on decision tree (Yosefian et al., 2015), which has good prediction accuracy without over-fitting, and it is suitable for survival analysis of many diseases (Farhadian et al., 2021; Roshanaei et al., 2022). The most important feature of the RSF algorithm is that it can rank the importance of variables in order to filter out those that have a greater impact on outcome indicators (Adham et al., 2017; Wang and Zhou, 2017). Moreover, RSF can effectively deal with the problem of data imbalance, when there is classification imbalance, RSF can balance the data error. Currently, it has been used in the construction of prognostic models for many different diseases, such as heart failure, arrhythmia, multiple myeloma, etc. (Hsich et al., 2011; Miao et al., 2015; Morvan et al., 2020). In addition, several studies have compared the performance of RSF with the classical Cox regression model, while some have found that RSF is more accurate than Cox regression (Sloan et al., 2016; Ma et al., 2020; Tapak et al., 2020), others have reached the opposite conclusion (Qiu et al., 2020). Hitherto, there were no comparative studies of two models in the ATN based on large sample data. Therefore, this study aimed to investigate the prediction of mortality in ATN patients in the ICU and the associated influencing factors by using RSF algorithm and Cox regression method.

## 2 Materials and methods

### 2.1 Data source and study population

Medical Information Mart for Intensive Care IV (MIMIC-IV) is a large-scale public database containing clinical information of patients at Beth Israel Deaconess Medical Center between 2008 and 2019, which was established by the Massachusetts Institute of Technology and Beth Israel Deaconess Medical Center. In the MIMIC-IV database, the patients’ true identifying information is hidden, therefore, there is no need to obtain informed consent from patients. However, researchers are required to complete relevant training courses and receive certificate before accessing the database. Datasets were obtained from the Physionet official website (http://mimic.physionet.org/).

A total of 4,031 patients were diagnosed with ATN in the database. For this study, the inclusion criteria were: over 18 years old and admission to the ICU longer than 24 h. Exclusion criteria were: patients who died within 24 h of the ICU admission and patients with incomplete data. For patients with multiple ICU admissions, and only data from their first admission were taken. Ultimately, a total of 3,220 patients were enrolled in this study.

### 2.2 Data extraction

Datasets were extracted by using structured query language. Basic information of ATN patients included: age at admission, gender, ethnicity, weight, length of stay in ICU, etc. Treatment measures included: antibiotic use, vasopressors use, nor-epinephrine use, the use of continuous renal replacement therapy, etc. Related comorbidities included the following: myocardial infarction, congestive heart failure, peripheral vascular disease, chronic pulmonary disease, cerebrovascular disease, rheumatic disease, mild liver disease, peptic ulcer disease, paraplegia, malignant cancer, severe liver disease, metastatic solid tumor, etc. The first laboratory test results after ICU admission included: hemoglobin, white blood cells, base excess, pH, anion gap (AG), bicarbonate, international normalized ratio (INR), prothrombin time, urine output, arterial partial pressure of oxygen, creatinine, BUN, chloride, glucose, etc. Vital signs after ICU admission included: heart rate, respiratory rate, systolic blood pressure (SBP), diastolic blood pressure (DBP), body temperature, etc. Because of the high sampling frequency, the maximum, the minimum and the average values were used to represent vital signs and laboratory test results.

### 2.3 Model construction

Patients with ATN were randomly divided into the training set and validation set in an 8:2 ratio. The training set was used to construct RSF or Cox model and the validation set was used to evaluate the performance of the two predictive models. RSF model was constructed on the basis of optimal parameter combination. The out-of-bag (OOB) error rate under different parameter combination, which is calculated by grid search method, and it was used to determine the optimal parameter combination of the model (Wang et al., 2019). RSF algorithm has the ability to assess the importance of each variable that contributed to the outcome indicators. In this study, the minimum depth method was used to measure and rank the importance of each variable (Peng et al., 2016). On the other hand, uni-variate and multivariate regression analysis were carried out for the Cox regression model. All variables were first analyzed in the uni-variate Cox regression model, and those with *p*-values less than 0.05 were selected and subjected to multivariate Cox regression analysis.

### 2.4 Model comparison

The OOB error rate is equivalent to the value of 1-C index, which is used to evaluate the prediction ability of the model. The smaller the out-of-bag error rate is, the stronger the differentiation ability of the model is. Brier score is an evaluation index to evaluate different survival models and can represent the prediction accuracy of prediction models. Brier score can be viewed as a “calibrated” measure of a set of probabilistic predictions. The OOB error rate and the integrated Brier score were first calculated to determine the discrimination and calibration ability of the two models. The smaller the OOB error rate, the better the discrimination ability of the predictive model (Banerjee et al., 2012). The model is well calibrated when the Brier score is less than 0.25 (Mogensen et al., 2012). And the smaller the Brier score, the better the calibration of the model. Then, the prediction error curves of two models were plotted for judging the prediction performance. To further assess the prognostic ability of two models, 30-day, 60-day and 90-day dependent receiver operating characteristic (ROC) curves were plotted. A larger area under the curve (AUC) value indicates a stronger predictive ability of the model when the AUC values are greater than 0.5 (Bansal and Heagerty, 2018). To analyze the decision curve analysis (DCA) of the Cox model, 30-day, 60-day, and 90-day DCA were plotted. When the net benefit of DCA is large, it indicates that the clinical application value of the model is high (Vickers and Holland, 2021). The superior performance model was utilized to construct a nomogram as individual prediction tool for ATN mortality risk.

### 2.5 Statistical analysis

For descriptive variables, the median and quartile were superior to the means and standard deviation values in several statistical guides (Kattan and Vickers, 2020). Therefore, continuous variables were represented by using the median and quartile and were compared by Mann-Whitney U test. Categorical variables were expressed in terms of frequency or percentage and compared using Chi-square tests or Fisher’s exact tests. In this study, indicators with the missing degree greater than 20% were removed, and then the remaining missing data were filled with multipe interpolation method (Zhang et al., 2022). Since the presence of outliers reduces the accuracy of RSF algorithm, the outliers were first identified by using box-plots, then the outlier indicators that exceed 10% were removed and median replacement was performed on the remaining outlier data (Dutta et al., 2022). The software packages used in the data analysis and processing included: randomForestSRC, survival, ggRandomForests, timeROC, and ggplot2.

## 3 Results

### 3.1 Baseline characteristics

A total of 3,220 patients with ATN were included in this study, of which 2,457 patients survived and 763 patients died during hospitalization. Comparisons between two groups showed that there were significant differences in the age, weight, length of stay in ICU, vasopressors, mild liver disease, severe liver disease, metastatic solid tumors, AG_min, AG_max, bicarbonate_min, bicarbonate_max, chloride_min, chloride_max, creatinine_min, creatinine_max, base excess_min, base excess_max, temperature_min, temperature_mean, urine output, DBP_min, etc. There were no statistically significant differences in the variables including gender, myocardial infarction, peripheral vascular disease, paraplegia, rheumatic disease, peptic ulcer disease, glucose_min, glucose_max, etc. Other baseline characteristics were shown in Tables 1–3.

**TABLE 1:** General information of the patients.

	Death	Survival	*p*-value
Number (sample size)	763	2,457	
Age,year	72.16(60.12,80.56)	66.32(54.98,77.06)	**< 0.0001**
Gender(%)			0.0529
Female	330(43.3)	966(39.3)	
Male	433(56.7)	1,491(60.7)	
Weight, kg	81(68.30,94.40)	83.2(70.20,98.80)	**0.0002**
Ethnicity (%)			**< 0.0001**
White	500(65.5)	1,647(67.0)	
Black	64(8.4)	296(12.0)	
Yellow	22(2.9)	65(2.6)	
Other	177(23.2)	449(18.4)	
Length of stay in the ICU, day	7.89(3.86,14.65)	5.80(2.78,12.21)	**< 0.0001**
First care unit (%)			**< 0.0001**
CCU	82(10.7)	311(12.6)	
SICU	118(15.5)	365(14.9)	
MICU	191(25.1)	715(29.1)	
CVICU	72(9.4)	295(12.0)	
Other	300(39.3)	771(31.4)	

*ICU*, intensive care unit; *CCU*, coronary care unit; *SICU*, surgical intensive care unit; *MICU*, medical intensive care unit; *CVICU*, cardiac vascular intensive care unit, *p*-value less than 0.05 are shown in bold text.

**TABLE 2:** The treatment and comorbidity of the patients.

	Death	Survival	*p*-value
*The treatment*			
Antibiotic (%)			**< 0.0001**
No	15(2.0)	158(6.4)	
Yes	748(98.0)	2,299(93.6)	
Dobutamine (%)			**0.0008**
No	707(92.7)	2,351(95.7)	
Yes	56(7.3)	106(4.3)	
Dopamine (%)			**0.0367**
No	700(91.7)	2,307(93.9)	
Yes	63(8.3)	150(6.1)	
Nerve blockers (%)			**< 0.0001**
No	671(87.9)	2,298(93.5)	
Yes	92(12.1)	159(6.5)	
Epinephrine (%)			**< 0.0001**
No	644(84.4)	2,263(92.1)	
Yes	119(15.6)	194(7.9)	
Norepinephrine (%)			**< 0.0001**
No	244(32.0)	1,388(56.5)	
Yes	519(68.0)	1,069(43.5)	
Phenylephrine (%)			**< 0.0001**
No	485(63.6)	1815(73.9)	
Yes	278(36.4)	642(26.1)	
Vasopressor (%)			**< 0.0001**
No	441(57.8)	2061(83.9)	
Yes	322(42.2)	396(16.1)	
CRRT(%)			0.3576
No	716(93.8)	2,327(94.7)	
Yes	47(6.2)	130(5.3)	
*Comorbidity*			
Myocardial infarct (%)			0.1509
No	576(75.5)	1916(78.0)	
Yes	187(24.5)	541(22.0)	
Congestive heart failure (%)			**0.0358**
No	415(54.4)	1,442(58.7)	
Yes	348(45.6)	1,015(41.3)	
Peripheral vascular disease (%)			0.9299
No	646(84.7)	2077(84.5)	
Yes	117(15.3)	380(15.5)	
Cerebrovascular disease (%)			**0.0047**
No	647(84.8)	2,178(88.6)	
Yes	116(15.2)	279(11.4)	
Dementia (%)			0.9653
No	735(96.3)	2,366(96.3)	
Yes	28(3.7)	91(3.7)	
Chronic pulmonary disease (%)			**0.0409**
No	521(68.3)	1772(72.1)	
Yes	242(31.7)	685(27.9)	
Rheumatic disease (%)			0.5712
No	730(95.7)	2,362(96.1)	
Yes	33(4.3)	95(3.9)	
Peptic ulcer disease (%)			0.7695
No	725(95.0)	2,341(95.3)	
Yes	38(5.0)	116(4.7)	
Mild liver disease (%)			**< 0.0001**
No	487(63.8)	1855(75.5)	
Yes	276(36.2)	602(24.5)	
Diabetes uncomplicated (%)			0.0777
No	564(73.9)	1735(70.6)	
Yes	199(26.1)	722(29.4)	
Diabetes complicated (%)			**0.0082**
No	658(86.2)	2018(82.1)	
Yes	105(13.8)	439(17.9)	
Paraplegia (%)			0.9334
No	738(96.7)	2,378(96.8)	
Yes	25(3.3)	79(3.2)	
Renal disease (%)			**0.0001**
No	496(65.0)	1,407(57.3)	
Yes	267(35.0)	1,050(42.7)	
Malignant cancer (%)			**< 0.0001**
No	553(72.5)	2,101(85.5)	
Yes	210(27.5)	356(14.5)	
Severe liver disease (%)			**< 0.0001**
No	597(78.2)	2,137(87.0)	
Yes	166(21.8)	320(13.0)	
Metastatic solid tumor (%)			**< 0.0001**
No	653(85.6)	2,334(95.0)	
Yes	110(14.4)	123(5.0)	
Aids (%)			0.3996
No	758(99.3)	2,431(98.9)	
Yes	5(0.7)	26(1.1)	

*CRRT*, continuous renal replacement therapy, *p*-value less than 0.05 are shown in bold text.

**TABLE 3:** Laboratory tests and vital signs of the patients.

	Death	Survival	*p*-value
*Laboratory tests*			
Hematocrit_min(%)	26.10(22.70,31.00)	27.00(23.40,32.00)	**0.0006**
Hematocrit_max(%)	31.20(27.20,35.85)	31.80(28.10,36.90)	**0.0004**
Hemoglobin_min(g/dL)	8.50(7.35,10.00)	8.80(7.70,10.50)	**< 0.0001**
Hemoglobin_max(g/dL)	10.10(8.80,11.60)	10.40(9.10,12.10)	**< 0.0001**
Platelets_min(k/uL)	138.00(69.0,203.5)	146.00(100.00,213.00)	**< 0.0001**
Platelets_max(k/uL)	183.00(105.00,247.00)	187.00(138.00,260.00)	**< 0.0001**
WBC_min(k/uL)	9.90(6.30,13.90)	9.90(6.70,13.30)	0.8786
WBC_max(k/uL)	13.80(9.40,19.30)	13.80(9.90,18.60)	0.9863
AG_min(mEq/L)	15.00(12.00,17.00)	14.00(12.00,17.00)	**0.0016**
AG_max(mEq/L)	19.00(16.00,22.00)	18.00(15.00,22.00)	**0.0114**
Bicarbonate_min(mEq/L)	18.00(15.00,22.00)	19.00(16.00,22.00)	**0.0003**
Bicarbonate_max(mEq/L)	22.00(19.00,25.00)	22.00(20.00,25.00)	**0.0007**
BUN_min(mg/dL)	33.00(21.00,50.50)	33.00(21.00,52.00)	0.7024
BUN_max(mg/dL)	41.00(26.00,59.50)	41.00(27.00,62.00)	0.2251
Calcium_min(EU/dL)	7.80(7.20,8.40)	7.80(7.30,8.40)	0.7065
Calcium_max(EU/dL)	8.40(8.0,9.0)	8.40(7.90,8.90)	0.1561
Chloride_min(mEq/L)	101.00(96.00,105.00)	101.00(97.00,106.00)	**0.0206**
Chloride_max(mEq/L)	105.00(100.00,110.00)	105.00(101.00,110.00)	**0.0202**
Creatinine_min(g/dL)	1.60(1.00,2.30)	1.70(1.20,2.60)	**< 0.0001**
Creatinine_max(g/dL)	2.00(1.30,3.00)	2.20(1.60,3.30)	**< 0.0001**
Sodium_min(mEq/L)	136.00(133.00,139.00)	136.00(133.00,139.00)	0.9822
Sodium_max(mEq/L)	140.00(136.00,143.00)	140.00(137.00,142.00)	0.9765
Potassium_min(mEq/L)	3.90(3.50,4.40)	4.00(3.60,4.40)	0.0801
Potassium_max(mEq/L)	4.60(4.10,5.20)	4.70(4.20,5.30)	0.0874
INR_min(s)	1.30(1.20,1.70)	1.30(1.10,1.50)	**< 0.0001**
INR_max(s)	1.50(1.40,2.00)	1.40(1.20,1.70)	**< 0.0001**
PT_max(s)	16.20(14.75,21.45)	15.70(13.40,18.90)	**< 0.0001**
PTT_min(s)	31.30(28.35,37.90)	30.30(26.90,34.20)	**< 0.0001**
pH_min	7.29(7.19,7.36)	7.30(7.22,7.36)	0.1004
pH_max	7.40(7.35,7.45)	7.40(7.35,7.45)	0.8361
PaO2_min(mmHg)	51.00(37.00,70.00)	53.00(39.00,79.00)	**< 0.0001**
PaO2_max(mmHg)	131.00(80.00,221.50)	134.00(79.00,243.00)	0.2706
PaCO2_min(mmHg)	33.00(28.00,39.00)	34.00(29.00,39.00)	**0.0035**
PaCO2_max(mmHg)	45.00(37.50,52.00)	45.00(39.00,52.00)	0.2290
TotalCO2_min	20.00(16.00,24.00)	21.00(17.00,24.00)	**0.0014**
TotalCO2_max	24.00(21.00,28.00)	25.00(22.00,28.00)	**0.0045**
Base excess_min(mEq/L)	−5.00(-11.00,-1.00)	−5.00(-9.00,-1.00)	**0.0033**
Base excess_max(mEq/L)	−1.00(-4.00,1.00)	−1.00(-4.00,1.00)	**0.0376**
Glucose_min(mg/dL)	108.00(86.50,132.00)	108.00(91.00,131.00)	0.1577
Glucose_max(mg/dL)	157.00(125.00,197.00)	157.00(127.00,204.00)	0.2225
Urine output(ml)	775.0(304.50,1392.50)	970.0(545.00,1732.00)	**< 0.0001**
*Vital Signs*			
Heart rate_min (min-1)	76.00(65.00,89.00)	73.00(63.00,84.00)	**< 0.0001**
Heart rate_max (min-1)	110.00(95.00,127.00)	104.00(90.00,120.00)	**< 0.0001**
Heart rate_mean (min-1)	91.41(80.08,103.60)	86.66(76.44,99.61)	**< 0.0001**
SBP_min(mmHg)	83.00(75.00,91.00)	85.00(78.00,96.00)	**< 0.0001**
SBP_max(mmHg)	139.0(125.00,152.00)	143.0(131.00,159.00)	**< 0.0001**
SBP_mean(mmHg)	106.73(99.52,116.70)	110.96(103.97,121.60)	**< 0.0001**
DBP_min(mmHg)	42.00(35.00,48.00)	44.00(38.00,50.00)	**< 0.0001**
DBP_max(mmHg)	83.00(71.75,96.00)	84.00(73.00,98.00)	**0.0249**
DBP_mean(mmHg)	58.20(52.01,64.63)	59.91(54.12,66.88)	**< 0.0001**
Respiratory rate_min (min-1)	13.00(11.00,16.00)	13.00(10.00,15.00)	**< 0.0001**
Respiratory rate_max(min-1)	30.00(26.00,35.00)	28.00(25.00,32.00)	**< 0.0001**
Respiratory rate_mean(min-1)	20.89(18.10,24.0)	19.64(17.31,22.52)	**< 0.0001**
Temperature_min(°C)	36.44(36.11,36.61)	36.44(36.17,36.67)	**0.0062**
Temperature_max(°C)	37.22(36.89,37.61)	37.22(36.94,37.61)	0.0505
Temperature_mean(°C)	36.78(36.51,37.03)	36.80(36.58,37.09)	**0.0002**
SpO2_min (%)	92.00(90.00,94.00)	92.00(90.00,95.00)	**0.0005**
SpO2_mean (%)	96.88(95.33,98.18)	97.00(95.66,98.44)	**0.0003**

*WBC*, white blood cells; *AG*, anion gap; *BUN*, blood urea nitrogen; *INR*, international normalized ratio; *PT*, prothrombin time; *PTT*, partial thromboplastin time, p*H* potential of hydrogen, *PaO2* partial pressure of oxygen, *PaCO2* partial pressure of carbon dioxide, *SBP*, systolic blood pressure; *DBP*, diastolic blood pressure, *SpO2* pulse oxygen saturation, *Max* maximum, *Min* minimum, *p*-value less than 0.05 are shown in bold text.

### 3.2 Model construction

#### 3.2.1 RSF model

**FIGURE 1:**
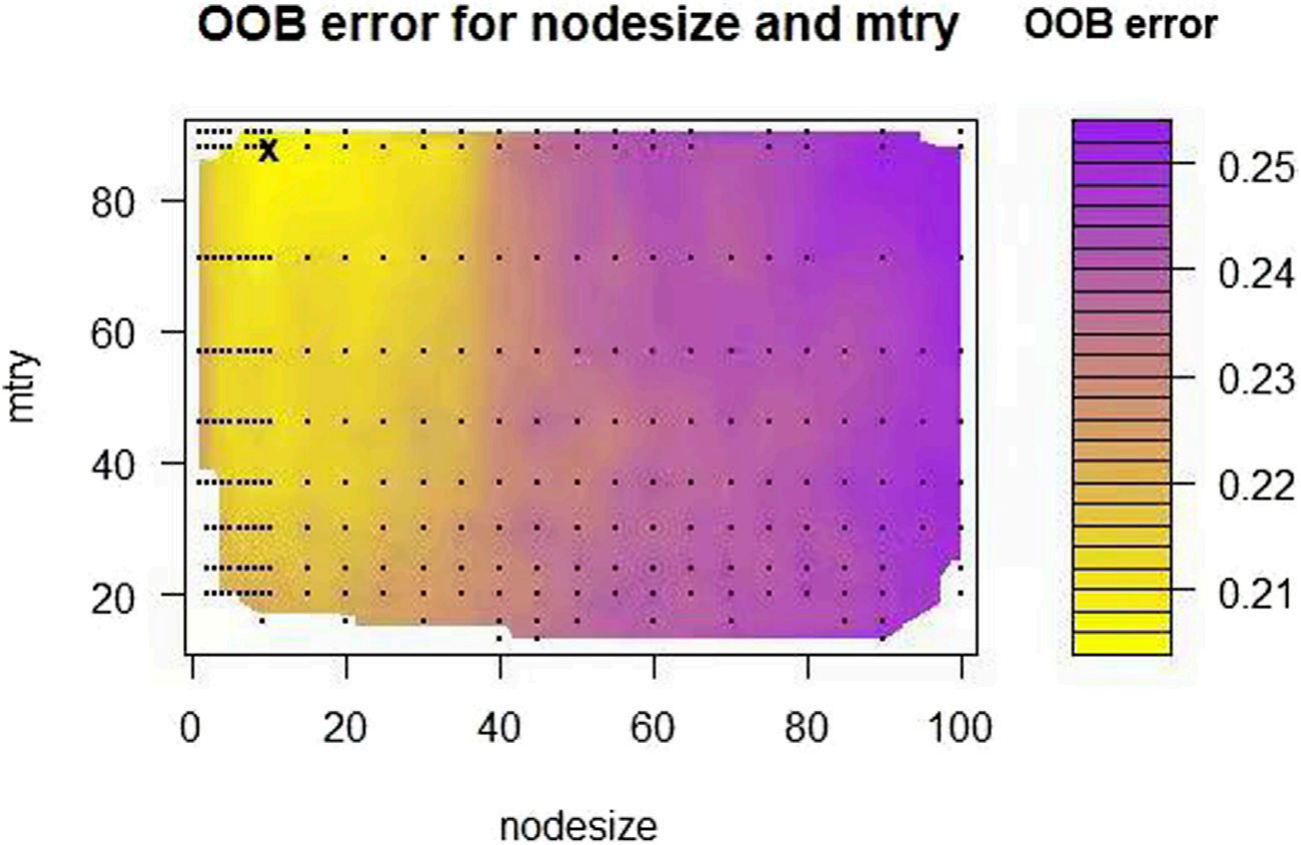
Tuning parameter of the RSF model. The black sign in the figure is the parameter combination of mtry = 10 and nodesize = 88, and the corresponding OOB error rate is 21.4%.

**FIGURE 2:**
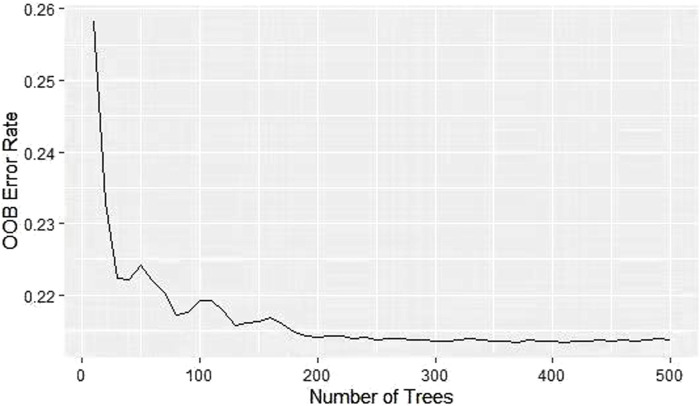
Curve of the OOB error rate for RSF model. The OOB error rate drops from 0.258 to 0.214 and stabilizes at 500 survival trees.

The OOB error rates were calculated by grid search method under different combination of parameters. As shown in Figure 1, RSF model achieved the lowest OOB error rate (21.4%) under the parameter combination of mtry = 10 and nodesize = 88. Different shades of the same color indicate the level of OOB error rate, while a shift from yellow to purple indicates an increase in the OOB error rate. The lower the OOB error rate, the better the predictive ability of model (Lines et al., 2021). As shown in Figure 2, the OOB error rate of model stabilized when 500 survival trees were reached. As shown in Table 4, the variables were ranked in importance by using the minimum depth method. Vasopressors, age, length of stay in ICU, metastatic solid tumors, INR_min, respiratory rate_min, chloride_max, calcium_min, base excess_min, bicarbonate_max, AG_min, potassium_min, pH_max, bicarbonate_min, DBP_min were the top 15 variables, indicating that these variables have strong predictive ability and significant effect on the outcome.

**TABLE 4:** Variable importance ranking for RSF.

Variable	Depth	Importance
Vasopressor	0.390	1
Age	1.364	2
Length of stay in the ICU	1.390	3
Metastatic solid tumor	3.460	4
INR_min	4.578	5
Respiratory rate_min	5.942	6
Chloride_max	6.430	7
Calcium_min	6.524	8
Base excess_min	7.050	9
Bicarbonate_max	7.248	10
AG_min	7.302	11
Potassium_min	7.874	12
pH_max	7.958	13
Bicarbonate_min	7.964	14
DBP_min	8.178	15
Calcium_max	8.214	16
SBP_min	8.452	17
WBC_max	8.550	18
Sodium_max	8.750	19
pH_min	8.882	20
PTT_min	8.964	21
Hemoglobin_min	9.132	22
Temperature_max	9.138	23
Weight	9.186	24
Chloride_min	9.202	25
Potassium_max	9.212	26
WBC_min	9.230	27
Hematocrit_min	9.788	28
Platelets_max	9.866	29
Platelets_min	9.886	30
PaO2_min	10.124	31
AG_max	10.312	32
PaCO2_min	10.364	33
Base excess_max	10.498	34
TotalCO2_max	10.596	35
BUN_min	10.684	36
Heart_rate_max	10.700	37
Creatinine_min	10.996	38
Glucose_min	11.004	39
SpO2_mean	11.166	40
Hemoglobin_max	11.384	41
Hematocrit_max	11.522	42
DBP_max	11.660	43
Sodium_min	11.750	44
Resp_rate_mean	11.896	45
BUN_max	12.012	46
PaCO2_max	12.194	47
Glucose_max	12.202	48
Urine output	12.324	49
Temperature_min	12.346	50
PaO2_max	12.964	51
Creatinine_max	12.998	52
Respiratory rate_max	13.392	53
SBP_max	13.650	54
SBP_mean	13.936	55
INR_max	13.996	56
Heart_rate_min	14.968	57
SpO2_min	15.236	58
DBP_mean	15.352	59

The 59 variables in the depth threshold and the corresponding minimum depth value; *ICU*, intensive care unit; *INR*, international normalized ratio; *AG*, anion gap, p*H* potential of hydrogen, *DBP*, diastolic blood pressure; *SBP*, systolic blood pressure; *WBC*, white blood cells; *PTT*, partial thromboplastin time, *PaO2* partial pressure of oxygen, *PaCO2* partial pressure of carbon dioxide, *BUN*, blood urea nitrogen, *SpO2* pulse oxygen saturation, *Max* maximum, *Min* minimum.

#### 3.2.2 Cox regression model

Uni-variate Cox regression analysis results revealed that variables included age, length of stay in ICU, dopamine, epinephrine, nor-epinephrine, vasopressors, AG_min, AG_max, bicarbonate_min, bicarbonate_max, INR_min, INR_max, pH_min, pH_max, base excess_min, base excess_max, urine output, SBP_min, SBP_max, SBP_mean, temperature_min, temperature_max, temperature_mean, cerebrovascular disease, chronic pulmonary disease, mild liver disease, severe liver disease, and metastatic solid tumors had *p*-value ≤0.05. These variables were included in the multivariate Cox regression model for analysis. Dopamine, epinephrine, nor-epinephrine, vasopressors, INR_min, cerebrovascular disease, chronic pulmonary disease, mild liver disease, severe liver disease, metastatic solid tumors were important risk factors that increased the risk of death in ATN patients. However, length of stay in ICU was protective factor. The shorter the stay, the lower the risk of death. Detailed information of Cox regression analysis was shown in Table 5.

**TABLE 5:** Cox regression analysis results.

Variable	Uni-variate Cox regression		Multivariate Cox regression	
	HR(95%CI)	*p*-Value	HR(95%CI)	*p*-Value
Age	1(1-1)	**< 0.0001**	1(1-1)	**< 0.0001**
Gender	0.82(0.7-0.97)	**0.02**	0.89(0.75-1.1)	0.2
Ethnicity	1.1(0.99-1.1)	0.095		
Weight	0.99(0.99-1)	**< 0.0001**	1(0.99-1)	0.095
First care unit	1(0.95-1.1)	0.76		
Length of stay in the ICU	0.96(0.95-0.97)	**< 0.0001**	0.95(0.94-0.95)	**< 0.0001**
Antibiotic	1.1(0.58-1.9)	0.85		
Dobutamine	1.3(0.96-1.8)	0.084		
Dopamine	1.4(1-1.8)	**0.037**	1.4(1-1.9)	**0.045**
Nerve blockers	1.2(0.91-1.5)	0.21		
Epinephrine	1.6(1.3-2)	**< 0.0001**	1.7(1.3-2.2)	**0.0002**
Norepinephrine	1.9(1.6-2.3)	**< 0.0001**	1.5(1.2-1.9)	**0.0004**
Phenylephrine	1.2(1-1.4)	**0.033**	1(0.86-1.3)	0.64
Vasopressor	2.4(2-2.8)	**< 0.0001**	2(1.6-2.5)	**< 0.0001**
CRRT	1(0.74-1.4)	0.89		
Hematocrit_min	1(1-1)	0.17		
Hematocrit_max	1(1-1)	0.2		
Hemoglobin_min	1(0.96-1)	0.77		
Hemoglobin_max	1(0.96-1)	0.9		
Platelets_min	1(1-1)	**0.027**	1(1-1)	0.99
Platelets_max	1(1-1)	**0.044**	1(1-1)	0.4
WBC_min	1(1-1)	0.13		
WBC_max	1(1-1)	0.12		
AG_min	1(1-1.1)	**< 0.0001**	0.99(0.96-1)	0.51
AG_max	1(1-1.1)	**< 0.0001**	1(0.98-1)	0.65
Bicarbonate_min	0.96(0.95-0.98)	**< 0.0001**	1(0.97-1)	0.68
Bicarbonate_max	0.96(0.94-0.98)	**< 0.0001**	1(0.96-1)	0.97
BUN_min	1(1-1)	0.58		
BUN_max	1(1-1)	0.98		
Calcium_min	0.93(0.85-1)	0.16		
Calcium_max	1(0.92-1.1)	0.71		
Chloride_min	1(0.99-1)	0.69		
Chloride_max	1(0.99-1)	0.86		
Creatinine_min	0.95(0.88-1)	0.2		
Creatinine_max	0.94(0.89-1)	0.061		
Glucose_min	1(1-1)	**0.044**	1(1-1)	0.25
Glucose_max	1(1-1)	0.14		
Sodium_min	1(0.99-1)	0.69		
Sodium_max	1(0.99-1)	0.18		
Potassium_min	0.95(0.84-1.1)	0.37		
Potassium_max	0.98(0.89-1.1)	0.73		
INR_min	2.1(1.7-2.6)	**< 0.0001**	1.5(1.1-2.1)	**0.0047**
INR_max	1.3(1.1-1.5)	**0.00054**	0.96(0.7-1.3)	0.78
PT_max	1(1-1)	**0.0013**	0.99(0.96-1)	0.44
PTT_min	1(1-1)	**< 0.0001**	1(1-1)	0.11
pH_min	0.38(0.19-0.77)	**0.0071**	0.71(0.21-2.4)	0.58
pH_max	0.21(0.07-0.62)	**0.0048**	2.4(0.44-13)	0.31
PaO2_min	0.99(0.99-1)	**< 0.0001**	1(0.99-1)	**0.049**
PaO2_max	1(1-1)	**0.0083**	1(1-1)	0.82
PaCO2_min	0.99(0.98-1)	**0.014**	1(0.99-1)	0.74
PaCO2_max	0.99(0.99-1)	0.05		
Base excess_min	0.98(0.96-0.99)	**0.00028**	1(0.98-1)	0.5
Base excess_max	0.96(0.94-0.98)	**< 0.0001**	0.99(0.95-1)	0.66
TotalCO2_min	0.97(0.96-0.99)	**0.00022**	1(0.98-1.1)	0.37
TotalCO2_max	0.96(0.95-0.98)	**< 0.0001**	0.97(0.94-1)	0.068
Urine output	1(1-1)	**< 0.0001**	1(1-1)	**0.0019**
Heart_rate_min	1(1-1)	0.13		
Heart_rate_max	1(1-1)	**0.024**	1(0.99-1)	0.9
Heart_rate_mean	1(1-1)	**0.039**	1(1-1)	0.13
SBP_min	0.98(0.98-0.99)	**< 0.0001**	1(1-1)	**0.021**
SBP_max	0.99(0.99-1)	**< 0.0001**	1(0.99-1)	0.84
SBP_mean	0.98(0.97-0.99)	**< 0.0001**	0.99(0.98-1)	0.28
DBP_min	0.97(0.96-0.98)	**< 0.0001**	0.99(0.98-1)	0.07
DBP_max	1(0.99-1)	0.19		
DBP_mean	0.98(0.97-0.99)	**< 0.0001**	1(0.99-1)	0.58
Respiratory rate_min	1(1-1.1)	**0.0033**	1(0.99-1.1)	0.11
Respiratory rate_max	1(1-1)	**0.00017**	1(0.99-1)	0.19
Respiratory rate_mean	1(1-1.1)	**0.00011**	1(0.98-1)	0.37
Temperature_min	0.78(0.66-0.91)	**0.0019**	0.84(0.68-1)	0.1
Temperature_max	0.85(0.74-0.97)	**0.019**	1.1(0.9-1.3)	0.38
Temperature_mean	0.75(0.63-0.9)	**0.0015**	0.9(0.67-1.2)	0.45
SpO2_min	0.97(0.95-0.99)	**0.0027**	1(0.97-1)	0.99
SpO2_mean	0.91(0.88-0.95)	**< 0.0001**	0.97(0.92-1)	0.25
Myocardial infarct	1.3(1.1-1.5)	**0.013**	1.1(0.88-1.3)	0.44
Congestive heart failure	1.2(0.98-1.4)	0.087		
Peripheral vascular disease	1.1(0.85-1.3)	0.6		
Cerebrovascular disease	1.3(1-1.6)	**0.038**	1.5(1.2-1.9)	**0.0007**
Dementia	1.6(1-2.4)	**0.041**	0.97(0.62-1.5)	0.9
Chronic pulmonary disease	1.3(1.1-1.5)	**0.012**	1.3(1.1-1.6)	**0.0071**
Rheumatic disease	1.4(0.96-2.1)	0.081		
Peptic ulcer disease	0.69(0.46-1)	0.08		
Mild liver disease	1.4(1.2-1.6)	**0.00022**	1.5(1.2-1.9)	**0.0003**
Diabetes uncomplicated	0.96(0.8-1.2)	0.7		
Diabetes complicated	0.83(0.65-1)	0.11		
Paraplegia	0.73(0.46-1.2)	0.17		
Renal disease	0.89(0.75-1.1)	0.2		
Malignant cancer	1.5(1.3-1.8)	**< 0.0001**	1.1(0.86-1.3)	0.59
Severe liver disease	1.2(1-1.5)	**0.045**	1.4(1-1.8)	**0.03**
Metastatic solid tumor	2.4(1.9-3.1)	**< 0.0001**	2.3(1.8-3.1)	**< 0.0001**
Aids	0.82(0.34-2)	0.66		

*HR*, hazard ratio; *CI*, confidence interval; *ICU*, intensive care unit; *CRRT*, continuous renal replacement therapy; *WBC*, white blood cells; *AG*, anion gap; *BUN*, blood urea nitrogen; *INR*, international normalized ratio; *PT*, prothrombin time; *PTT*, partial thromboplastin time, p*H* potential of hydrogen, *PaO2* partial pressure of oxygen, *PaCO2* partial pressure of carbon dioxide, *SBP*, systolic blood pressure; *DBP*, diastolic blood pressure, *SpO2* pulse oxygen saturation, *Max* maximum, *Min* minimum, *p*-value less than 0.05 are shown in bold text.

### 3.3 Model comparison

**FIGURE 3:**
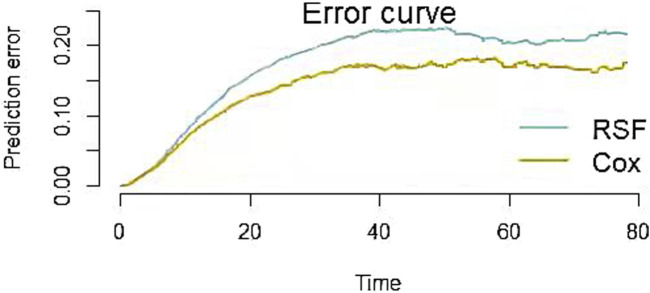
Prediction error curves for RSF and Cox models. The smaller the prediction error value, the more accurate the predictive power of the model.

**FIGURE 4:**
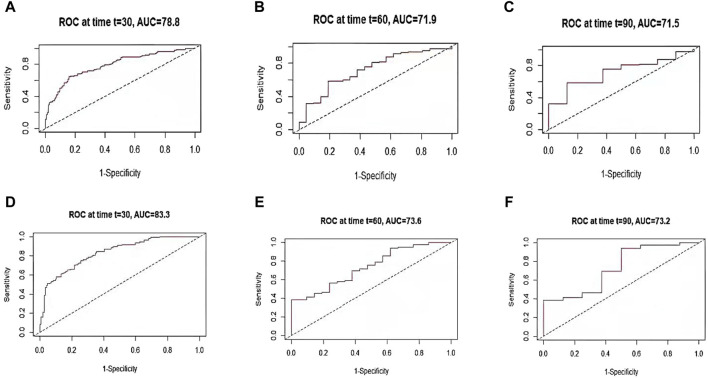
TdROC curve for RSF and Cox models. The AUC values for RSF model at 30-day **(A)**, 60-day **(B)**, and 90-day **(C)** are 0.788, 0.719, and 0.715, respectively; the AUC values for Cox regression model at 30-day **(D)**, 60-day **(E)**, and 90-day **(F)** are 0.833, 0.736, and 0.732, respectively.

**FIGURE 5:**
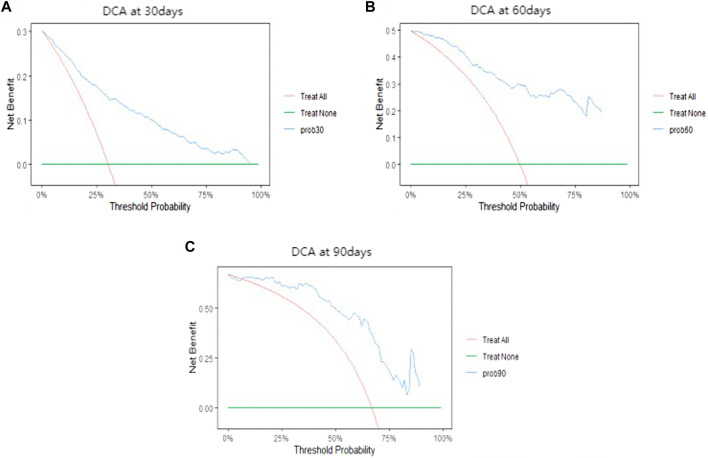
Decision curve analysis of the Cox model.

As shown in Table 6, the OOB error rates for RSF model and Cox regression model were 0.214 and 0.215. The OOB error rates for two models were small, indicating that both predictive models had good discrimination ability. The integrated Brier score of two models were 0.199 (RSF) and 0.154 (Cox), both of which were less than 0.25, suggesting that two models had good calibration ability. As shown in Figure 3, the prediction error curve of Cox regression model was lower than that of RSF model, indicating that Cox regression model was more stable and reliable. In the validation set, RSF model had AUC values of 0.788, 0.719, and 0.715 at 30, 60 and 90 days (Figure 4). Cox regression model had AUC values of 0.833, 0.736, and 0.732 at 30, 60 and 90 days (Figure 4). Thus, it appeared that Cox regression model was more accurate in predicting mortality of ATN patients. As shown in Figure 5, the net benefit of Cox model at different time points was large, indicating that the Cox model had high clinical practical value.

**TABLE 6:** Performance comparison of the two models.

Model	OOB	IBS
Random survival forest	0.214	0.199
Multivariate cox regression	0.215	0.154

*OOB*, out-of-bag error rate; *IBS*, integrated brier score.

### 3.4 Nomogram for predicting risk of ATN mortality

**FIGURE 6:**
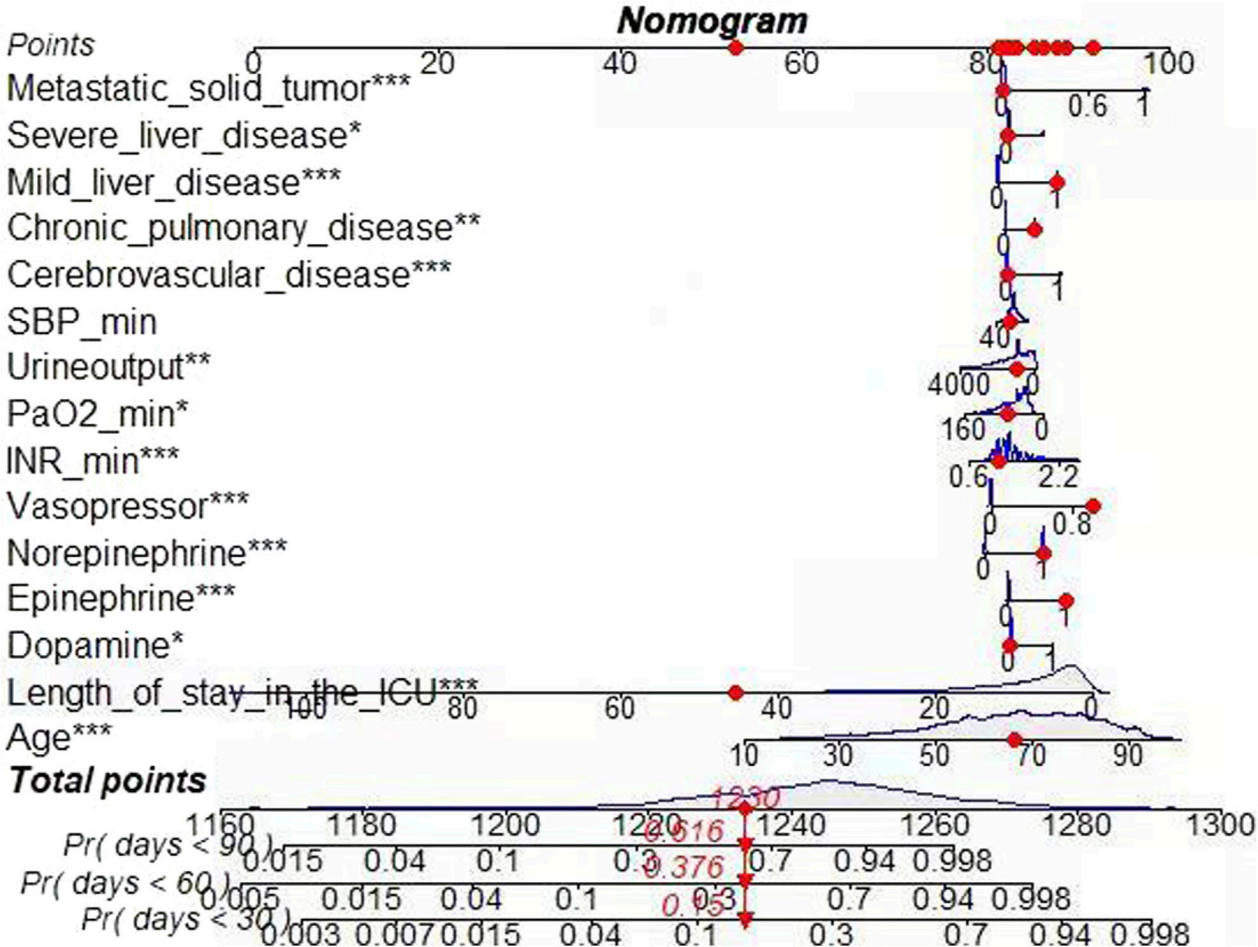
Nomogram for Cox regression model. Risk of death: 0.15 (30 days); 0.376 (60 days); 0.616 (90 days).

Given that Cox model outperforms RSF model in the discrimination and calibration ability, a nomogram was constructed on the basis of Cox model to predict the probability of death at the individual level in ATN patients. The nomogram was constructed to predict 30-day, 60-day, and 90-day mortality risk based on 15 significant variables in the training set. In the nomogram, an individual score for each factor is obtained by projecting the value of each factor vertically onto the first row of “points”. For each participant, the total score was calculated by adding the scores for each factor. By projecting the total score vertically to the bottom, we can get a picture of the risk of death for ATN patients. Assuming a 68-year-old patient with ATN has a score of 82 for metastatic solid tumor, 83 for severe liver disease, and 87 for mild liver disease. Chronic pulmonary disease score was 84, cerebrovascular disease score was 82, SBP_min score was 83, urine output score was 83, and PaO2_min score was 82. INR_min score was 80, vasopressor score was 91, nor-epinephrine score was 86, epinephrine score was 88, dopamine score was 81, length of stay in the ICU score was 55, with an age score of 83 out of 1,230, the estimated risk of death at 30, 60, and 90 days was 15%, 37.6%, and 61.6% (Figure 6).

## 4 Discussion

ATN is one of the most common types of acute kidney injury, which seriously affects the quality of life and even threatens their lives, and it is characterized by high morbidity, high mortality and poor prognosis (Hoste et al., 2018). Therefore, it is important to identify the influencing factors for ATN, which can help screen those patients at high risk and receive proper treatment.

Although ML methods represented by RSF algorithm performed well in several fields, this does not mean that MLalgorithms have an absolute advantage over traditional methods. For example, Cuthbert et al. (Cuthbert et al., 2022) analyzed the prediction of 8-year revision risk following total knee and hip arthroplasty, and Tang X et al. (Tang X. et al., 2021) studied prognostic prediction in metastatic non-small cell lung cancer patients receiving EGFR-TKI osiertinib treatment, both studies have proved that traditional statistical methods have certain strengths. Nonetheless, RSF algorithm also has its own unique advantages. RSF algorithm can directly and quantitatively calculate the minimum depth of each variable to reflect the magnitude of importance, which facilitates the comparison among variables (Taylor, 2011). This is a feature which has not been found in the traditional Cox regression method. Therefore, it is better to construct predictive models by using several methods and compare them to identify the best ones.

Since Cox model outperforms RSF model, we constructed a nomogram based on the Cox model. The nomogram is a visualization tool used to generate the probability of clinical outcome (Park, 2018). Studies have demonstrated that nomogram enable accurate compared to traditional scoring systems (Wang et al., 2021). It is now widely used for risk prediction of many diseases (Li et al., 2021; Tan et al., 2022; Wang et al., 2023). Therefore, the creation of mortality risk nomogram based on the information about ATN patients can inform existing critical care assessment programs. Clinical staff can use the total score to predict the probability of death in ATN patients, thus assisting them in developing a more rational treatment plan.

In this study, two different models were constructed to explore the influencing factors of ATN. Cox regression analysis concluded that vasopressors, nor-epinephrine, INR_max, severe liver disease, and metastatic solid tumors were the important risk factors. RSF model concluded that vasopressors, INR_max, and metastatic solid tumors were the important influencing factors based on the importance rank of the variables. The influencing factors identified by two methods of analysis are basically similar, indicating that they are probably true factors associated with ATN.

Among the variables associated with predicting ATN patients, the most important ones are the AG, pH, base excess, BUN and bicarbonate, which can be used to determine whether patients have symptoms of acid-base imbalance, azotemia and electrolyte disturbances (Bellomo, 2011). Urine output is the most common factor affecting ATN. This is due to decreased urine output can cause hypovolemia, which increases the risk of death from the disease (Xu et al., 2020). Timely rehydration therapy can restore the circulating blood volume and improve the impaired renal perfusion function.

ATN is often associated with many comorbidities, and the presence of these comorbidities also increases the risk of death from ATN. Severe liver disease is a relatively common comorbidity in patients with ATN. Due to the presence of large amounts of peritoneal fluid in patients with severe hepatitis, it can lead to insufficient circulating blood volume and uneven distribution of intrarenal blood flow, which ultimately increases the probability of death in ATN (Chancharoenthana and Leelahavanichkul, 2019). In addition, metastatic solid tumors are the common comorbidity that increases the risk of death from this disease (Wu et al., 2020), and the main reason is that neutrophils in solid tumors can enhance cytotoxicity and lead to necrosis of renal tubular epithelial cells, thus reducing patient survival (Liao and Liaw, 2020).

Relevant studies have shown that certain drugs also increase the risk of death in ATN. For example, vasopressor drugs can increase glomerular perfusion pressure and urine output, thus affecting renal function (Shi and Wang, 2017). Nor-epinephrine can increase patients’ blood pressure and reduce renal blood flow, resulting in renal function impairment (Kim et al., 2021). INR is a preferred monitoring indicator for oral anticoagulants. Since overdose of anticoagulants increases the probability of death from ATN, the level of this indicator also reflects the risk of death occurring from ATN (Lim and Campbell, 2013).

However, the association between these drugs and disease needs further study. Because machine learning is one of the main methods of drug knowledge discovery. In the follow-up study, we plan to use machine learning, text mining and other technical methods to mine the process of drug tacit knowledge contained in the data, so as to explore whether there is a potential association between drugs and some biomedical entities, such as drug-disease association, and the association between drugs and side effects, etc.

The main advantage of this study is that it was the first to use the RSF algorithm and the Cox regression method to predict hospital mortality of patients with ATN from the MIMIC-IV database. Cox regression model has improved accuracy and precision compared to RSF model. This study also has some limitations: firstly, it is a single-center study and lacks external validation. Secondly, this was a retrospective observational study in which the majority of patients were white, and there may have been unobserved confounding factors that could have influenced the outcome. Finally, although the predictive ability of Cox regression model in this study is superior toRSF model, ML algorithms are evolving rapidly and new algorithms are constantly proposed, and further comparative studies are needed in practical applications.

## 5 Conclusion

Cox regression model is superior to RSF algorithm model in predicting mortality of patients with ATN. Vasopressors, nor-epinephrine, INR_min, and metastatic solid tumors were imporant factors that also significantly influence prognosis. Therefore, the mortality risk nomogram based on information about ATN patients can inform existing critical care assessment programs. Moreover, the model has certain clinical utility, which can provide clinicians with some reference basis in the treatment of ATN and contribute to improve patient prognosis.

## Figures and Tables

**Table:** 

**ATN**	Acute tubular necrosis
**RSF**	Random survival forest
**OOB**	Out-of-bag
**ICU**	Intensive care unit
**pH**	Potential of hydrogen
**BUN**	Blood urea nitrogen
**ML**	Machine learning
**MIMIC**	Medical information mart for intensive care
**AG**	Anion gap
**INR**	International normalized ratio
**SBP**	Systolic blood pressure
**DBP**	Diastolic blood pressure
**ROC**	Receiver operating characteristic
**AUC**	Area under the curve
**Max**	Maximum
**Min**	Minimum
**CCU**	Coronary care unit
**SICU**	Surgical intensive care unit
**MICU**	Medical intensive care unit
**CVICU**	Cardiac vascular intensive care unit
**CRRT**	Continuous renal replacement therapy
**WBC**	White blood cells
**PT**	Prothrombin time
**PaO2**	Partial pressure of oxygen
**PTT**	Partial thromboplastin time
**PaCO2**	Partial pressure of carbon dioxide
**SpO2**	Peripheral capillary oxygen saturation
**HR**	Hazard ratio
**CI**	Confidence interval

## Data Availability

The datasets presented in this study can be found in online repositories. The names of the repository/repositories and accession number(s) can be found below: https://physionet.org/content/mimiciv/1.0/.
